# The Impact and Implications of Regenerative Medicine in Urology

**DOI:** 10.7759/cureus.52264

**Published:** 2024-01-14

**Authors:** Abdullah I Abuharb, Abdullah F Alzarroug, Saad N Algahtani, Hatan K Alghamdi, Fahad A Alosaimi, Nasser Alsuwayna, Alwaleed I Almughira

**Affiliations:** 1 College of Medicine, Imam Mohammad Ibn Saud Islamic University, Riyadh, SAU

**Keywords:** genitourinary surgery., safety, efficacy, urology, regenerative medicine

## Abstract

Urology focuses on the treatment of genitourinary disorders through therapies ranging from lifestyle changes to advanced surgeries; the field has recently incorporated robotic and minimally invasive technologies that have improved patient outcomes and reduced hospital stays and complications. However, these methods still have certain limitations. Regenerative medicine, focusing on natural repair abilities, can be an effective and safer alternative. This review aims to examine the impact of regenerative medicine in urology.

We adopted a systematic review design by following the Preferred Reporting Items for Systematic Reviews and Meta-Analyses (PRISMA) guidelines. An exhaustive online literature search involving the databases PubMed, the Cochrane Central Register of Controlled Trials (CENTRAL), and Google Scholar was conducted spanning the period between January 2010 and October 2023. Data were extracted from studies on regenerative medicine in urology with a special focus on efficacy and safety.

Data from 16 studies were analyzed, which showed that cell therapy, biological materials, and tissue engineering are generally used in the field of urinary diseases. The main applications include the regeneration of urinary tissue, the correction of urinary incontinence, the treatment of erectile dysfunction, the reconstruction of ureteric defects, and the formation of bladder tissue. The study findings generally lack definitive conclusions on effectiveness and safety. While our results indicate that regenerative medicine is successful on a subjective level, more clinical trials are needed to establish its effectiveness and safety.

## Introduction and background

Urology is a medical field that specializes in treating genitourinary disorders, ranging from simple lifestyle changes to more invasive and advanced surgical procedures. In recent decades, this field has witnessed significant technological advancements through the introduction of cutting-edge robotics and minimally invasive surgeries, significantly improving patient outcomes and prognosis, and reducing overall hospital stay and postoperative complications. However, the literature continues to show certain limitations, especially in terms of traditional management options. These options may require extended use of medications, associated with temporary relief or a high risk of side effects [1,2].

Regenerative medicine is an innovative and rapidly developing treatment strategy in various medical disciplines [3]. It emphasizes enhancing the body's natural ability to heal and regenerate injured tissues and organs, as well as promoting tissue repair and functional recovery [4]. These strategies include stem cell therapy, tissue engineering, and the delivery of growth factors [5]. Currently, urology is undergoing a significant transformation in terms of the formulation of therapies related to regenerative medicine. The integration of this approach could revolutionize disease management, reducing safety concerns and increasing efficiency. Recent studies have shown that bladders grown in laboratories can be successfully transplanted and that urological tissues can be rebuilt [6]. The evidence of its efficacy and clinical applications has mushroomed, highlighting the transformative potential of regenerative medicine in urology.

Given the rapid increase in interest in the applications of regenerative medicine in the field of urology and the huge uptick in research activities, it is necessary to comprehensively assess the current state of the evidence. This systematic review aims to review the current state and progress of research in regenerative medicine and assess its significance and impact on urology.

## Review

Methodology

Study Design

This is a systematic review conducted based on the Preferred Reporting Items for Systematic Reviews and Meta-Analyses (PRISMA) guidelines.

Literature Search

PubMed, the Cochrane Central Register of Controlled Trials (CENTRAL), and Google Scholar were used to search for relevant studies on the topic spanning the period between January 2010 and October 2023. Keyword combinations, Boolean operators, MeSH terms, field tags, and truncations were used to identify studies. "Urology" AND "regenerative medicine" AND "treatment" were the primary keywords used in the search. The following MeSH terms were also used: (("Urologic Diseases/therapy"[Mesh] AND "Tissue Engineering [Mesh]) OR "Stem Cell Transplantation"[Mesh] OR "Regenerative Medicine"[Mesh].

Eligibility Criteria

The studies were chosen based on the established eligibility criteria, using the PECOS framework. The primary inclusion criterion was as follows: studies involving individuals diagnosed with a genitourinary disease. Research that evaluated human and animal participants, through in vivo or in vitro methods, was considered eligible for inclusion. The article had to involve the exposure of participants to regenerative medicine modalities as the primary intervention. The criteria did not insist on the use of a specific comparator (C); however, controlled trials were permitted. The primary outcome (O) of interest was the conclusion of the experiment and the urological application of the regenerative modality. The accepted study designs (S) included prospective observation studies and clinical trials. 

Data Extraction

Data from the chosen papers was extracted on a standardized Microsoft Excel sheet. Data related to study authors, publication year, outcome of interest, number of subjects, type of observation, type of regenerative medicine, follow-up period, urological applications, and conclusive conclusions were extracted.

Results 

Study Selection

**Figure 1:**
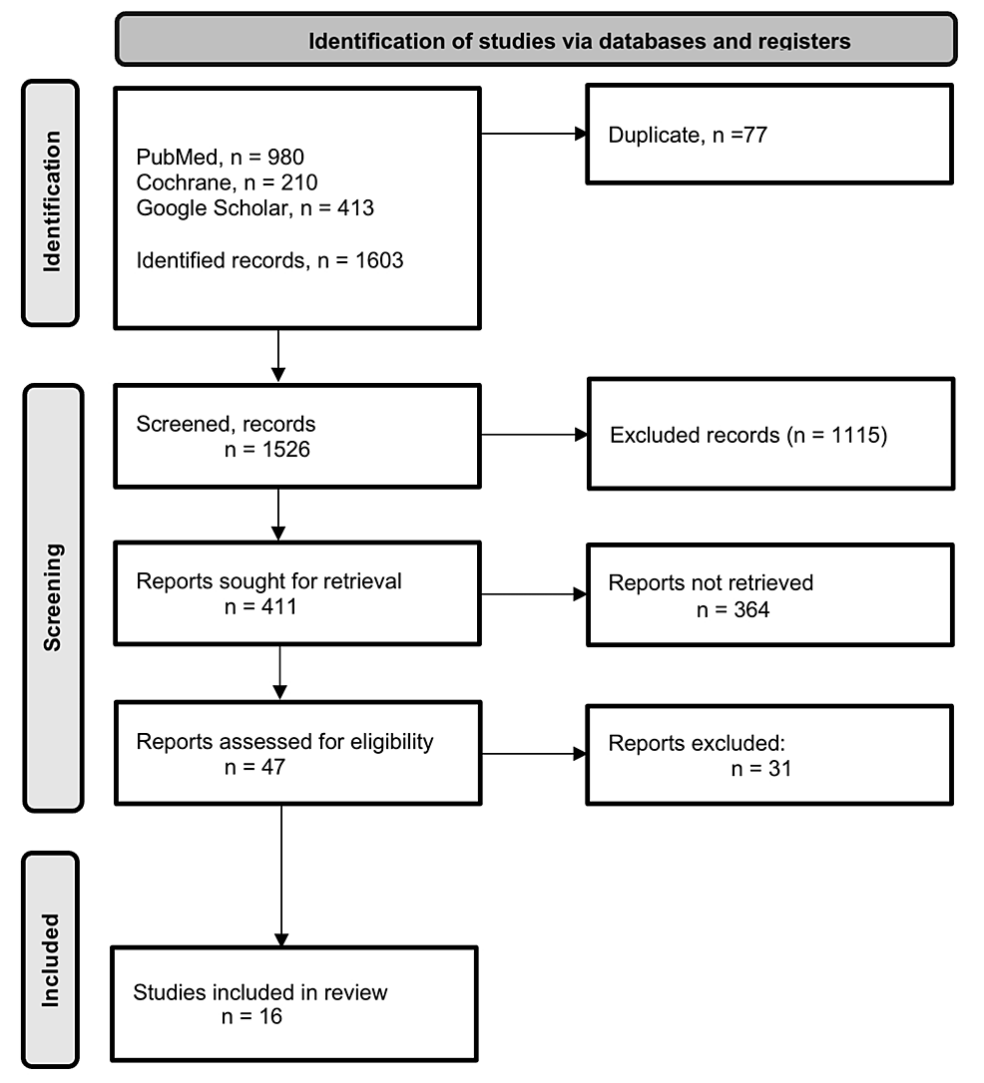
PRISMA flowchart depicting the selection of studies PRISMA: Preferred Reporting Items for Systematic Reviews and Meta-Analyses

A total of 1603 studies were identified from online database searches (PubMed: 980, Google Scholar: 413, and CENTRAL: 210). Duplicates and automated filters removed 77 studies, leaving 1,526 for screening. Title and abstract screening eliminated 1115, leaving 411. Full-text screening removed 364, while 47 studies advanced to methodological screening and further full-text screening. After excluding 31 studies, this systematic review ultimately included only 16 that met the inclusion criteria. Figure 1 below shows the PRISMA flow diagram depicting the study selection process.

Study Characteristics

Table 1 provides a summary of the characteristics of the included studies.

**Table 1:** Characteristics of the selected articles ADRC: adipose-derived regenerative cell; ADSC: adipose tissue-derived stem cell; BC: bacterial cellulose; CDI: carbodiimide; CNS: cavernous nerve stimulation; cGMP: cyclic guanosine monophosphate; ECM: extracellular matrix; eNOS: endothelial nitric oxide synthase; ED: erectile dysfunction; GP: genipin; ICP: intracranial pressure; MAP: mean arterial pressure; nNOS: neuronal nitric oxide synthase; PD: Peyronie's disease; PEU: polyesterurethane; SMC: smooth muscle cell; SVF: stromal vascular fraction; SUI: stress urinary incontinence; TA: tunica albuginea; UDSC: urine-derived stem cell; VEGF: vascular endothelial growth factor

Study	Type of regenerative medicine	Urological application	Conclusion
Morgante et al., 2021 [7]	Biomaterials	Urethra to treat hypospadias	Evidence that implanted non-crosslinked acellular matrices readily incorporate to support surgical repair. Acellular matrix onlay grafts enhance repair quality and reduce complications
Orabi et al., 2013 [8]	Biomaterials	Urethral reconstruction	Preclinical evidence of cell-seeded tubularized scaffolds for reconstructing long urethral defects. Bladder-derived acellular collagen matrix with autologous cells led to normal urethral tissue development over time
Raya-Rivera et al., 2011 [9]	A tissue biopsy	Urethral reconstruction	Urethral biopsies revealed a normal architecture 3 months post-implantation. Tubularized urethras can be engineered and remain functional for as long as six years in a clinical setting
Garcia-Arranz et al., 2020 [10]	Mesenchymal stem cells	Treatment of urinary incontinence	A 70% to 80% subjective improvement from baseline. No adverse effects were observed. Intraurethral application of stem cells derived from adipose tissue is a safe and feasible treatment for postradical prostatectomy or female SUI
Gotoh et al., 2013 [11]	ADRCs	Treatment of SUI	There was a 59.8% decrease in leakage volume in the 24 h pad test. The mean maximum urethral closing pressure and functional profile length increased from 35.5 to 44.7 cmH_2_O and 20.4 to 26.0 mm, respectively. Periurethral autologous ADRC injection is a safe and feasible treatment for male SUI, and likely for female SUI as well
Yamamoto et al., 2012 [12]	Autologous adipose tissue-derived regenerative cells	SUI	Maximum urethral closing pressure and functional profile length increased; progressive increase in blood flow to the injected area. No significant adverse events were observed. Urinary incontinence improved from two weeks post-injection up to six months
Gokce et al., 2014 [13]	ADSCs	Prevention and treatment of ED	Significantly higher ICP/MAP and total ICP in response to cavernous nerve stimulation CNS. Local ADSC injection prevented/reduced Peyronie's-like changes. Research confirms ADSC benefits on penile fibrosis and erectile function
Castiglione et al., 2012 [14]	ADSCs	ADSCs on improving erectile function	Erectile function significantly improved with ADSC treatment. PD animals' fibrosis and elastosis areas were prevented by ADSC treatment. ADSC injection prevents fibrosis and elastosis in the TA and corpus cavernosum
Huang et al., 2010 [15]	ADSCs	Treatment of ED	ADSC ameliorates nerve and endothelial abnormalities, promising a potential therapy for ED
Das et al., 2014 [16]	Stem and stromal cells	Treating ED	Human SVF treatment significantly increased cavernous endothelial and smooth muscle cell contents, induced eNOS phosphorylation, and restored penile nNOS-positive nerve fibers. Erectile function significantly improved in diabetic mice treated with human SVF and SVF lysate
Ryu et al., 2012 [17]	SVF from epididymal adipose	Restoration erectile function	SVF increased cavernous endothelial cell proliferation, eNOS phosphorylation, and cGMP expression. SVF promotion of cavernous angiogenesis and erectile function was abolished with VEGF-Trap, a VEGF-A neutralizing antibody
Bodin et al., 2010 [18]	UDSCs	Urinary bladder reconstruction	Porous BC scaffolds enable 3D USC growth, forming a multilayered urothelium and cell-matrix infiltration. Cell-seeded BC scaffolds hold promise for tissue-engineered urinary conduits in urinary reconstruction
Horst et al., 2015 [19]	Hybrid microfibrous PEU and poly lactic-co-glycolic acid scaffolds	Bladder tissue formation	PEU-hybrid scaffolds promote bladder tissue formation with excellent integration and low inflammation. PEU is a promising biomaterial for tissue engineering
Adamowicz et al., 2020 [20]	A novel biocomposite	Urinary bladder wall regeneration	The graphene layer significantly increased biocomposite electrical conductivity. The graphene layer efficiently stimulated SMC with a strong cell-to-biomaterial interface
Zhao et al., 2020 [21]	Differentiated human-USCs	Ureter reconstruction	Ultimately, a layered ureter structure with multilayered urothelium over organized smooth muscle tissue. Tissue-engineered graft formed multilayered urothelium and organized smooth muscle tissue after ureteral reconstruction
Koch et al., 2015 [22]	Decellularized ureters	Reconstructing ureteric defects	In vitro: CDI and genipin GP scaffolds had more ingrown 3T3 and rat SMCs than untreated scaffolds. In vivo: implants were mainly infiltrated by fibroblasts and M2 anti-inflammatory macrophages. CDI was the most beneficial for crosslinking ECM scaffolds. Results aid in developing a biocompatible ureteral xenograft

Discussion

Tissue engineering involves the utilization of bioengineering principles and biomaterials to create a viable biological transplant for normal tissue and its associated functionality [23]. Using autologous tissues for reconstructive surgeries may carry an additional risk of graft rejection, and sequelae of postoperative immunosuppressive therapy [24]. On the other hand, clinical follow-ups have shown delayed healing of the area of the donor’s body from where the tissue was removed [25]. Tissue engineering offers a promising alternative for the restoration of damaged tissues and organs associated with the field of urology. Over the last two decades, there has been a notable increase in scientific interest in stem cells and their capacity for regeneration and differentiation.

Biomaterials and Tissue Engineering

Biomaterials provide physical adhesion to the extracellular matrix (ECM). In regeneration, biomaterials regulate cell differentiation, migration, proliferation, and gene expression [26]. Morgante et al. investigated whether decellularized tissue matrices could recreate urological tissue's physiologic and biochemical functions. Ideally, biomaterials facilitate tissue growth with minimal adverse effects. The paper also proposed biomaterials such as urethral grafts for tissue integration and cellularization. All porcine survived surgery and 2.5 years of postoperative surveillance without any issues. The implantation of decellularized tissue matrices in large animal surgery models is well-tolerated and non-inflammatory [7].

Orabi et al. documented novel biomaterials for urethral repair in biocompatible tubularized tissue constructs. Traditionally, tissues from the buccal mucosa, bladder mucosa, or genital flaps were used to repair defects such as strictures and increased urethral caliber. Orabi et al. introduced cell-seeded tubularized scaffolds for wider caliber urethras. Within 12 months, the cellular organization increased, an epithelial cell layer formed, and muscle fiber bundles formed in collagen scaffolds. Seeded cells labeled with a fluorescent marker were monitored for three months. Urothelial and smooth muscle cells survived, proliferated, and contributed to the multilayered tissue structure, confirming the tissue-engineering potential of cell-seeded biomaterials [8].

Seeding autologous cells promotes tissue integration and minimizes inflammation. This is supported by Horst et al., who used hybrid PEU scaffolds to form acellular bladder matrices and encourage the formation of bladder tissue with a low inflammatory response. The researchers investigated a property of biomaterials used in regenerative modalities. Implanted biomaterials mimic target tissue properties for similar regenerated tissues. Horst et al. used high-elasticity PEU hybrid scaffolds, similar to a urinary bladder, to transmit mechanical forces to regenerate smooth muscle cells [19].

To mimic the bladder's neuronal network, Adamowicz et al. added graphene to an amniotic membrane biocomposite to increase electrical conductivity. A biocompatible graphene layer increased electrical conductivity, resembling a bladder wall, without cytotoxicity for smooth muscle cells [27]. Graphene-based scaffolds may help tissue engineering restore organ function [28]. The literature endorses the use of graphene-layered biomaterials for muscle differentiation and proliferation [29,30], which warrants preclinical in vivo trials.

Cell-Based Therapies

Under certain conditions, stem cells can differentiate into specialized cells. Stem cells include embryonic stem cells (ESCs), induced pluripotent stem cells (iPCSs), somatic stem cells, fetal stem cells, cord blood stem cells, perinatal stem cells, cancer stem cells (CSCs), and multipotent/unipotent stem cells [31]. They have a wide range of applications in tissue engineering, regenerative medicine, and cell therapy, particularly as autologous stem cells derived from an individual's body. For instance, adult mesenchymal stem cells (MSCs) offer therapeutic potential through differentiation and lifelong organ regeneration [13,32].

Adipose tissue-derived stem cells (ADSCs) are effective in the treatment of several medical conditions, and their immunosuppressive properties have been acknowledged by An et al [32]. According to the Association for the Advancement of Blood and Biotherapies, transplanted cells can heal spinal cord injuries, joint cartilage, neurologic disorders, and the immune system. Silverman et al. modified patient or donor cells to overcome diseases and medical conditions, with advances like chimeric antigen receptor therapy for blood cancers [33]. However, safety, tumorigenicity, and high manufacturing costs make cell-based therapies difficult to implement. Despite these barriers, cell-based therapies offer unique advantages, as cells can naturally migrate, localize, and proliferate in specific tissues or compartments [34].

Garcia-Arranz et al. demonstrated the safety of autologous MSCs from liposuction. Patients were evaluated every three months for one year after endoscopic intraurethral injection. This alternative therapy aimed to treat urinary incontinence, with lower associated risks compared to traditional treatments [10]. The small sample size limits conclusive effectiveness assessment, building on earlier studies like Huang et al. [10,15]. In vitro, multipotent ADSCs from adipose tissue can differentiate into neuron-like, endothelial, and smooth muscle cells. Huang et al. autologously injected cultured ADSCs into the corpus cavernosum of 18 rats to increase smooth muscle content promise for erectile dysfunction treatment [15]. Das et al. reproduced similar results in male mice, with stem and stromal cells from human breast adipose tissue-derived stromal vascular fraction (SVF). Stem cells differentiated to increase cavernous endothelial and smooth muscle cell content, restoring erectile function significantly [16]. Bone marrow-derived MSCs secrete neurotrophic factors that induce neural regeneration, as seen in VEGF, brain-derived neurotrophic factors, and nerve growth factors [35].

ADSCs and human SVF cells were found in small amounts in the corpus cavernosum 14 days after implantation [15,16]. Other investigators observed rapid stem cell disappearance at around four weeks, suggesting migration and death rather than differentiation [36]. Current evidence suggests that stem cells migrate to the bone marrow due to their nature [37-39]. Cell-based therapies rely on various cell types for seeding therapeutic scaffolds to induce tissue regeneration. ADSCs, human SVF cells, and urine-derived stem cells (USCs) have been studied for tissue-engineered organs. In vitro, Bodin et al. used bacterial cellulose (BC) scaffolds seeded with USC to regenerate urinary diversion conduits with multilayers of smooth muscle and urothelial cells over two weeks [18]. In athymic mice requiring cystoplasty, differentiation into smooth muscle cells and urothelial markers was observed in vivo [18]. Multiple in vitro regeneration and in vivo implantation attempts have shown that dynamic cultures promote cell growth and muscle and urothelial layer formation [19,22,40]. Bodin et al. also noted an increased cellular infiltration into the bacterial cellulose matrix in dynamic cultures compared to static cultures [18,40]. Koch et al. crosslinked scaffolds with various agents and incubated them for two weeks before implanting them to reconstruct the urinary bladder [18,27] or the ureters [21,22]. This showed constructive remodeling and integration of the scaffold into the surrounding tissue.

Clinical Outcomes and Safety

All studies in this review explored regenerative therapies for urologic conditions, including urethral tissue regeneration [21], urinary incontinence correction [11,12], erectile dysfunction treatment [14,17], ureteric defect reconstruction, and bladder tissue formation [22]. Overall, these 16 studies suggest that regenerative medicine effectively builds new tissues to address the target condition. Some studies even replicated the neurological network of the rebuilt tissues, indicating the reliability of regenerative medicine [14-16]. Tissue innervation is crucial for replicating the original tissue's properties in the regenerated tissues, particularly in addressing conditions like erectile dysfunction [16]. However, assessing the effectiveness of regenerative medicine has certain limitations. Current literature uses small sample sizes, in vitro experiments, and non-human subjects, making it difficult to assess its efficacy [10]. According to Ławkowska et al., advanced reconstructive urology techniques outperform regenerative techniques in clinical settings, casting doubt on the widespread use of regenerative medicine in urology [41]. The primary issue involves the inability to accurately replicate the complex native tissue environment. On reviewing the evidence, regenerative medicine appears to be most successful in treating urinary incontinence [10-12], but the clinical trials that arise from this evidence show short-term success compared to placebo treatments [42,43]. 

Safety concerns in regenerative medicine have not yet raised significant alarms on a small scale. Koch et al. found no cytotoxic effect with the implants and Yamamoto et al. reported safe peri-urethral injection of regenerative cells [12,22]. However, the discourse on the safety of regenerative medicine is multifaceted and dependent on therapy, cell source, tissue type, and administration method. For instance, Zhou et al. noted a potential link between VEGF, which promotes cell regeneration, and cancer development [43], indicating that long-term carcinogenic risk may exist within biomaterials [41,44]. Current safety evidence is highly subjective to individual studies, which necessitates further large-sample clinical trials and comprehensive meta-analyses.

## Conclusions

This review examined the current state of regenerative medicine, with a focus on its uses in urology. Regenerative medicine is a cutting-edge field that combines biology, medicine, and engineering, with the main objective of repairing, replacing, or regenerating human cells, tissues, or organs. This technique has the potential to greatly improve the treatment of diseases of the urinary and reproductive systems in urology. Our findings illustrate the great advances that regenerative medicine has brought to the field of urology. These studies showcase a variety of cutting-edge methods, such as tissue engineering for urethral reconstruction, gene editing for the treatment of prostate cancer, and stem cell therapy for bladder dysfunctions, which could usher in a paradigm shift in urological care, away from traditional symptomatic therapy and toward treating the underlying causes of illnesses.

Regenerative medicine holds promise for treating renal problems, incontinence, and erectile dysfunction in urology. Instead of only treating symptoms, these therapies try to return the body to its normal state. However, there are often unanticipated side effects that emerge during clinical studies, making the transition from research to therapeutic use difficult; these highlight the need to comprehend the long-term effects and interactions with the reproductive and urinary systems. Despite these drawbacks, regenerative therapy in urology holds great promise. It might completely change the way many urological illnesses are treated by providing more potent and possibly even curative treatments. However, to fully exploit this promise, comprehensive and carefully monitored clinical trials are required to determine the safety and efficacy of these novel medicines, which would shed on light the complexities of regenerative treatments within the urological setting and guarantee compliance with strict regulatory requirements.

In urology, regenerative medicine is still in a phase of development; therefore, it is critical to maintain a balance between innovation and safety and ethical issues. Along with overcoming scientific and medical obstacles, the industry also needs to deal with accessibility, ethical, and legal concerns. Regenerative medicine in urology has a bright future ahead of it, with potentially ground-breaking therapies that could improve patient outcomes and quality of life.
